# Supplementary material for: Schwann cells, but not Oligodendrocytes, Depend Strictly on Dynamin 2 Function
Source: eLife. 2019 Jan 16;8:e42404. doi: 10.7554/eLife.42404 (PMC6335055; doi:10.7554/eLife.42404)
Supplement: Supplementary file 1. [file elife-42404-supp1.docx]

**
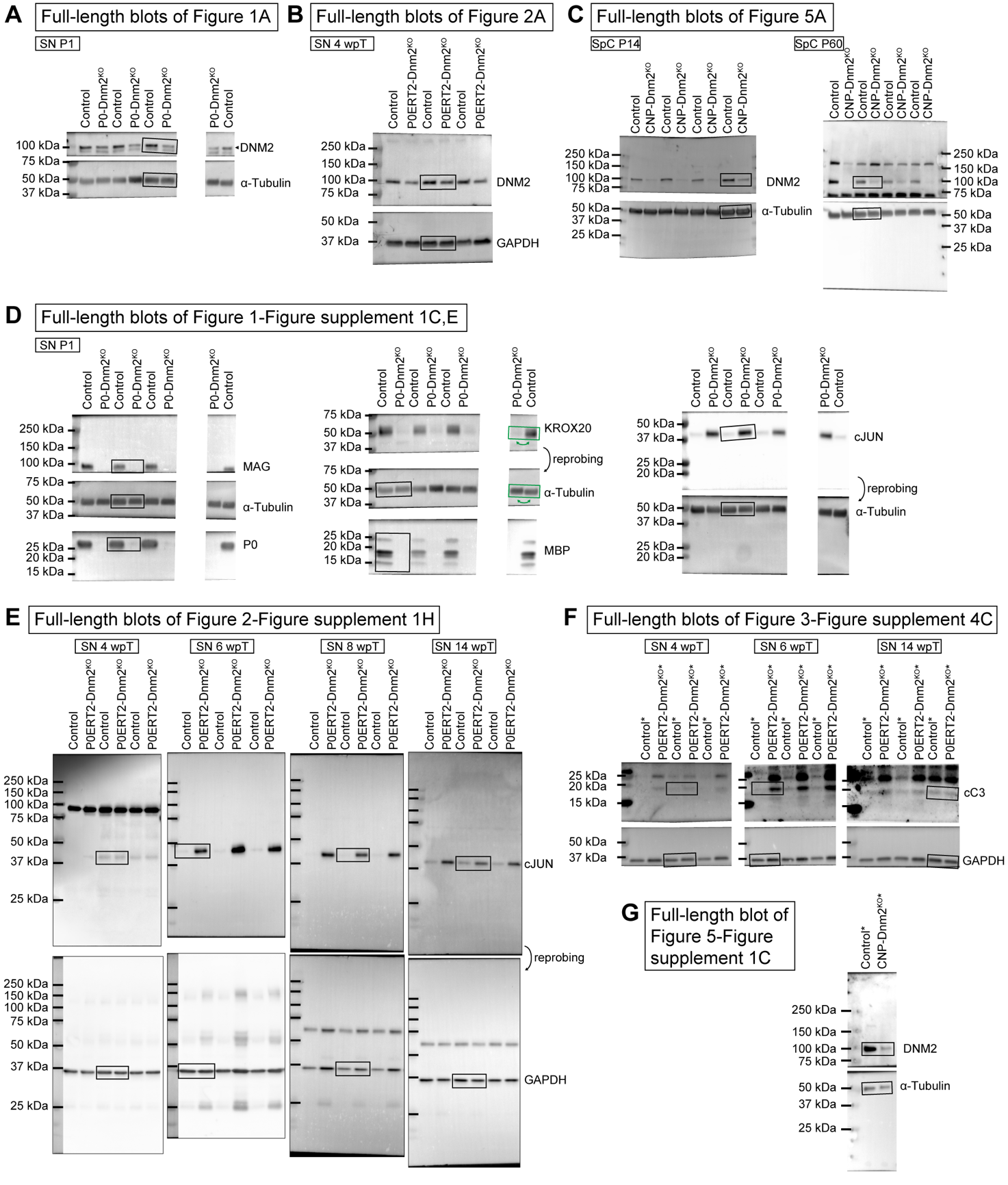
Supplementary File 1.** Full-length western blot images

The western blots are overlaid with the corresponding membranes. The membrane was cut and probed with the indicated antibodies. Black rectangles indicate region selected for the figures. The molecular weights refer to the Precision Plus Protein Standards (#161-0373, BioRad).

**A)** Western blot in Figure 1A; quantification in Figure 1-Figure supplement 1B.

**B)** Western blot in Figure 2A; quantification in Figure 2-Figure supplement 1B.

**C)** Western blot in Figure 5A; quantification in Figure 5-Figure supplement 1B.

**D)** Western blot in Figure 1-Figure supplement 1 C, E; quantification in Figure 1-Figure supplement 1D,F.

**E)** Western blot in Figure 2-Figure supplement 1H; quantification in Figure 2-Figure supplement 1I.

**F)** Western blot in Figure 3-Figure supplement 4C; quantification in Figure 3-Figure supplement 4D.

**G)** Western blot and quantification in Figure 5-Figure supplement 1C.
